# zAMP and zAMPExplorer: reproducible scalable amplicon-based metagenomics analysis and visualization

**DOI:** 10.1093/bioadv/vbaf255

**Published:** 2025-11-04

**Authors:** Valentin Scherz, Sedreh Nassirnia, Farid Chaabane, Violeta Castelo-Szekely, Gilbert Greub, Trestan Pillonel, Claire Bertelli

**Affiliations:** Institute of Microbiology, Lausanne University Hospital and University of Lausanne, 1011 Lausanne, Switzerland; Institute of Microbiology, Lausanne University Hospital and University of Lausanne, 1011 Lausanne, Switzerland; Institute of Microbiology, Lausanne University Hospital and University of Lausanne, 1011 Lausanne, Switzerland; Institute of Microbiology, Lausanne University Hospital and University of Lausanne, 1011 Lausanne, Switzerland; Institute of Microbiology, Lausanne University Hospital and University of Lausanne, 1011 Lausanne, Switzerland; Institute of Microbiology, Lausanne University Hospital and University of Lausanne, 1011 Lausanne, Switzerland; Institute of Microbiology, Lausanne University Hospital and University of Lausanne, 1011 Lausanne, Switzerland

## Abstract

**Summary:**

To enable flexible, scalable, and reproducible microbiota profiling, we have developed zAMP, an open-source bioinformatics pipeline for the analysis of amplicon sequence data, such as 16S rRNA gene for bacteria and archaea or ITS for fungi. zAMP is complemented by two modules: one to process databases to optimize taxonomy assignment, and the second to benchmark primers, databases and classifier performances. Coupled with zAMPExplorer, an interactive R Shiny application that provides an intuitive interface for quality control, diversity analysis, and statistical testing, this complete toolbox addresses both research and clinical needs in microbiota profiling.

**Availability and implementation:**

Comprehensive documentation and tutorials are provided alongside the source code of zAMP and zAMPExplorer software to facilitate installation and use. zAMP is implemented as a Snakemake workflow, ensuring reproducibility by running within Singularity or Docker containers, and is also easily installable via Bioconda. The zAMPExplorer application, designed for visualization and statistical analysis, can be installed using either a Docker image or from R-universe.

## 1 Introduction

The microbiota is a dynamic and complex ecological community, essential to ecosystem balance and functionality. In human and veterinary medicine, increasing evidence supports its role in health and disease (Hou *et al.* 2022, Scherz *et al.* 2022b). Beyond ecological insights, microbiota profiling also offers possibilities for novel diagnostic and prognostic tools with potential to improve clinical decisions (Malla *et al.* 2018). Targeted metagenomics (metabarcoding) by high-throughput sequencing of the *16S rRNA* encoding gene and the Internal Transcribed Spacer (ITS) regions are routinely used to characterize bacterial, archaeal, and fungal communities (Armougom 2009). It complements traditional methods in clinical microbiology offering high sensitivity and broad species detection, particularly in samples harboring complex communities such as polymicrobial abscesses, outperforming culture (Gupta *et al.* 2019, Nam *et al.* 2023).

However, analyzing microbiomes remains challenging due to the sample diversity, the compositional complexity of microbial communities, and inconsistencies in analytical and statistical methodologies (Knight *et al.* 2018, Trinh *et al.* 2018, Scherz *et al.* 2022a). These challenges complicate the identification of microbial taxa associated with health outcomes, interventions, or forensic investigations (Trinh *et al.* 2018, Gouello *et al.* 2021). Additionally, biases at every step from library preparation to bioinformatics analysis affect data accuracy and interpretability (Pfeifer 2017, McLaren *et al.* 2019, Nearing *et al.* 2021). Bioinformatics pipelines are particularly sensitive to parameters and even minor changes in clustering algorithms, classifiers, or database selection significantly influence reproducibility and reliability (Siegwald *et al.* 2017, Kulkarni *et al.* 2018, Nearing *et al.* 2018, Ye *et al.* 2019). These issues underscore the importance of standardized, rigorous protocols and bioinformatic pipelines across research, clinical, and forensic applications (Pollock *et al.* 2018, Fjukstad *et al.* 2019, Scherz *et al.* 2022a).

Commercial microbiome analysis platforms such as EzBiome (Yoon *et al.* 2017), Illumina’s BaseSpace Sequence Hub (Illumina 2024), One Codex (Minot *et al.* 2015), and QIAGEN Microbial Genomics Module (QIAGEN Digital Insights 2001) offer comprehensive workflows with various features tailored for both clinical and research applications. Yet, they are paid services and require data submission to tertiary infrastructure for cloud computing, limiting accessibility for certain users (Stajich and Lapp 2006, Marizzoni *et al.* 2020, Mbareche *et al.* 2020, Prodan *et al.* 2020). Open-source projects, like QIIME2 (Caporaso *et al.* 2010, Bolyen *et al.* 2019), and MOTHUR (Schloss *et al.* 2009) provide tutorials and tools for microbiota profiling. However, these tools can be challenging to install and use without bioinformatics expertise and may lack reproducibility and flexibility to accommodate novel workflows or specific experimental needs (Dudley and Butte 2009, Lutz *et al.* 2022). Furthermore, many pipelines do not address the limitations of 16S rRNA amplicon sequencing, such as its inability to distinguish closely related taxa due to highly conserved sequences in the targeted regions (Johnson *et al.* 2019).

In-house bioinformatics pipelines present an attractive alternative, offering adaptable solutions that benefit from community-driven open-source development (Fjukstad *et al.* 2019, Mbareche *et al.* 2020, Straub *et al.* 2020, Scherz *et al.* 2022a). Workflow management systems like Snakemake (Mölder *et al.* 2021) or Nextflow (Di Tommaso *et al.* 2017) improve usability and efficiency by automating complex analyses and parallelizing processes (Caporaso *et al.* 2010).

In this setting, we have developed zAMP, an open-source Snakemake-based bioinformatics pipeline designed for scalability and ease of use. zAMP simplifies microbiota profiling through a single-command installation, introduces a database pre-processing step to improve taxonomic classification accuracy, and integrates a wide range of standard amplicon analysis tools. Additionally, zAMPExplorer, an R Shiny app (R Core Team 2014, Jackson *et al.* 2021), streamlines data exploration and visualization to facilitate the analysis and interpretation of microbiota profiling from fundamental research to clinical applications.

## 2 Implementation of zAMP, a reproducible, scalable, amplicon-based metagenomics pipeline

**Figure 1.**
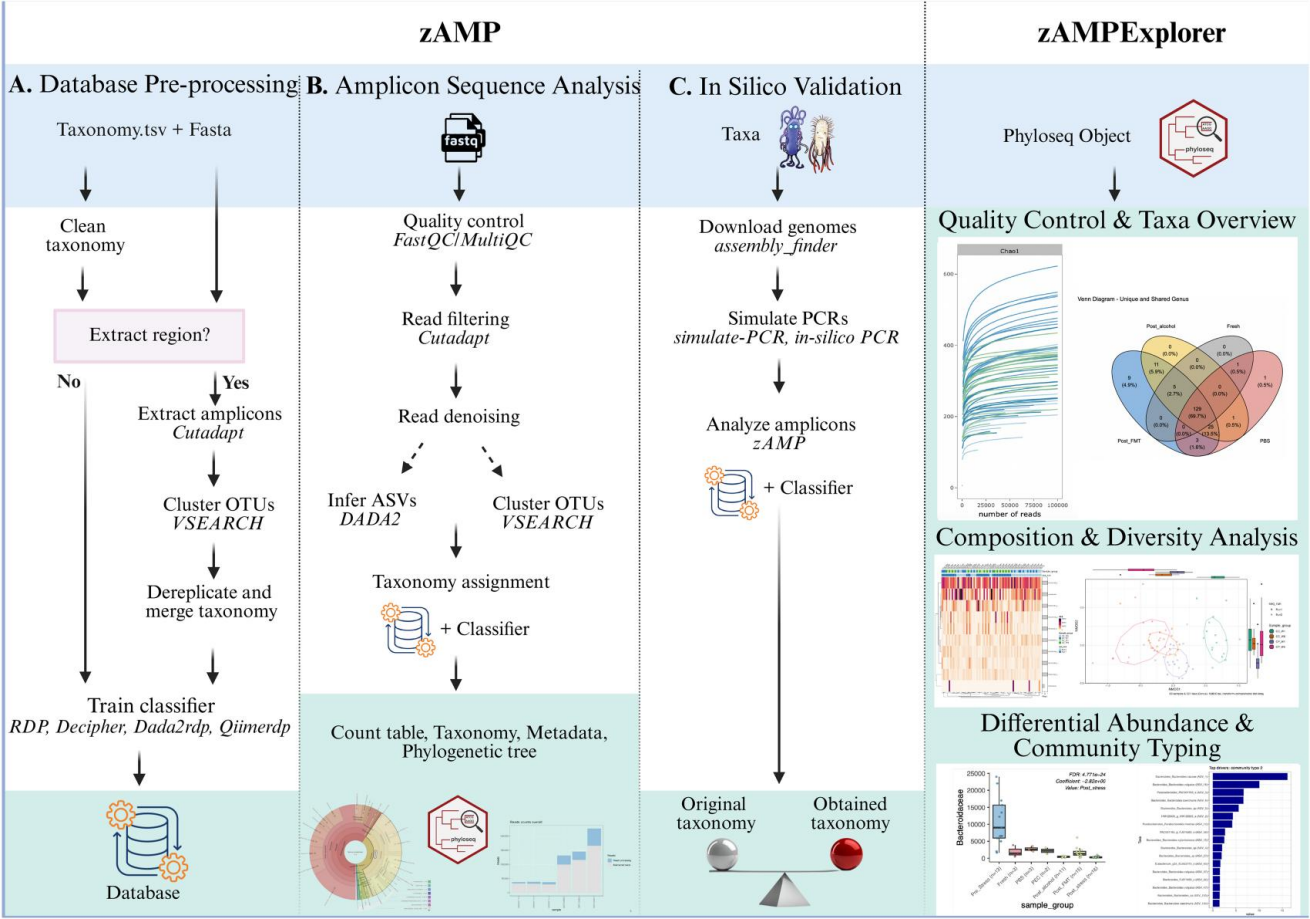
zAMP and zAMPExplorer modules. zAMP includes three modules: (A) to pre-process the reference database, and merge taxonomic ranks that cannot be distinguished based on the target region. This module has been tested with EzBioCloud, SILVA, Greengenes2, UNITE, and Eukaryome databases. (B) To analyze amplicon sequences and assign taxonomic ranks. (C) To assess the classification of known genomes to evaluate the tool accuracy for specific microorganisms, given the target genomic region. zAMPExplorer, an R Shiny app, allows to visualize a phyloseq object and perform quality control and compositional analyses. The blue section represents the inputs, the green section represents the outputs, and the white section outlines the workflow and processing steps, with third party tools in italics. Created in BioRender. Nassirnia *et al.* (2025)  https://BioRender.com/v06h290.

zAMP was developed to simplify amplicon sequence analysis through a single command line (Fig. 1). Sensible default parameters can be adjusted, providing flexibility to meet diverse research and clinical needs. Users can set forward and reverse primer sequences, adjust minimum read length for read quality control, set denoiser for Operation Taxonomic Unit (OTU) or Amplicon Sequence Variant (ASV) inference and perform taxonomic assignment with classifiers such as RDP (Wang *et al.* 2007, Wang and Cole 2024), assignTaxonomy (Callahan *et al.* 2016), and q2-feature-classifier (Bokulich *et al.* 2018), respectively. This adaptability enables tailoring to specific study design and data requirements. zAMP integrates state-of-the art software selected for being open-source, published in a peer-reviewed journal and containerized to ensure reliability and reproducibility. Pipeline version, tool command lines and parameters are logged for full traceability. Additionally, database files created by zAMP are assigned a unique hash to ensure consistency and reproducibility.

zAMP accepts as input local reads in fastq format or SRA accessions to directly download reads via the SRA Toolkit (Leinonen *et al.* 2011). Next, reads containing forward or reverse primers are extracted with Cutadapt, trimmed and quality filtered with DADA2 (Callahan *et al.* 2016) filterAndTrim function. By default, reads passing quality control are denoised and chimeras are removed to output ASVs. zAMP also offers an alternative denoising method with VSEARCH (Rognes *et al.* 2016), which clusters sequences at ≥97% identity into OTUs. The OTU approach reduces the interpretation of sequencing errors as biological variants but cannot resolve fine-scale variations important to differentiate some commensal or pathogenic strains (Allard *et al.* 2015, Callahan *et al.* 2016, Fasolo *et al.* 2024, Fares *et al.* 2025). To assign sequences a taxonomy, zAMP uses by default the naïve Bayesian classifier from RDP (Wang *et al.* 2007, Wang and Cole 2024), or alternatively DADA2 (Callahan *et al.* 2016), QIIME 2 (Dubois *et al.* 2022), Sintax (Edgar 2016), Kraken2 (Wood et al. 2019), and Decipher (Murali *et al.* 2018). Finally, zAMP can rarefy samples at a user-defined threshold to mitigate biases from uneven sampling depth (Sanders 1968). Outputs are standardized (e.g. tab-delimited ASV/OTU tables, and BIOM files) and consolidated into a single phyloseq object (McMurdie and Holmes 2013), facilitating data management and analysis in R.

Quality control is pivotal to detect wet-lab or sequencing failure as well as unsuitable parameters that may yield spurious results (Scherz *et al.* 2022a). zAMP incorporates FastQC (Andrews 2010) for sample-level quality assessment of sequences and MultiQC (Ewels *et al.* 2016) for aggregated run quality report. The pipeline generates tables, bar plots and Krona plots to track read counts through processing steps and explore sample composition. Rarefaction curves are provided to assess sequencing depth adequacy. With its streamlined, one-command execution, zAMP simplifies microbiome data analysis.

zAMP has been successfully applied to diverse sample types, from environmental to veterinary and human specimens (stools, urines, throat swabs, sputa, saliva, and sterile fluids) (Bozza *et al.* 2022, Scherz *et al.* 2022a, Galperine *et al.* 2023, Kritikos *et al.* 2025, Nassirnia *et al.* 2025). It supports various primer sets, target regions (e.g. ITS or 16S rRNA), and taxonomic databases, making it suitable for use both in research and in diagnostics.

## 3 zAMP benchmarking

To contextualize zAMP’s utility, we compared its resource usage and taxonomic assignment accuracy to its closest open-source alternative nf-core/Ampliseq (Straub *et al.* 2020). Both use workflow management systems and perform amplicon sequence analysis via a single command. We simulated V3–V4 amplicon reads at 30× coverage from a reference dataset of 1196 pathogenic species identified at Lausanne University Hospital (Table 1, available as supplementary data at *Bioinformatics Advances* online) using MeSS (Chaabane *et al.* 2024). We generated subsets of 25, 50, 100, 200, 400, 800, 1000, and 1196 species, each with five replicates. We processed the simulated reads using zAMP and ampliseq, and collected runtime metrics via an automated Nextflow pipeline.

**Figure 2.**
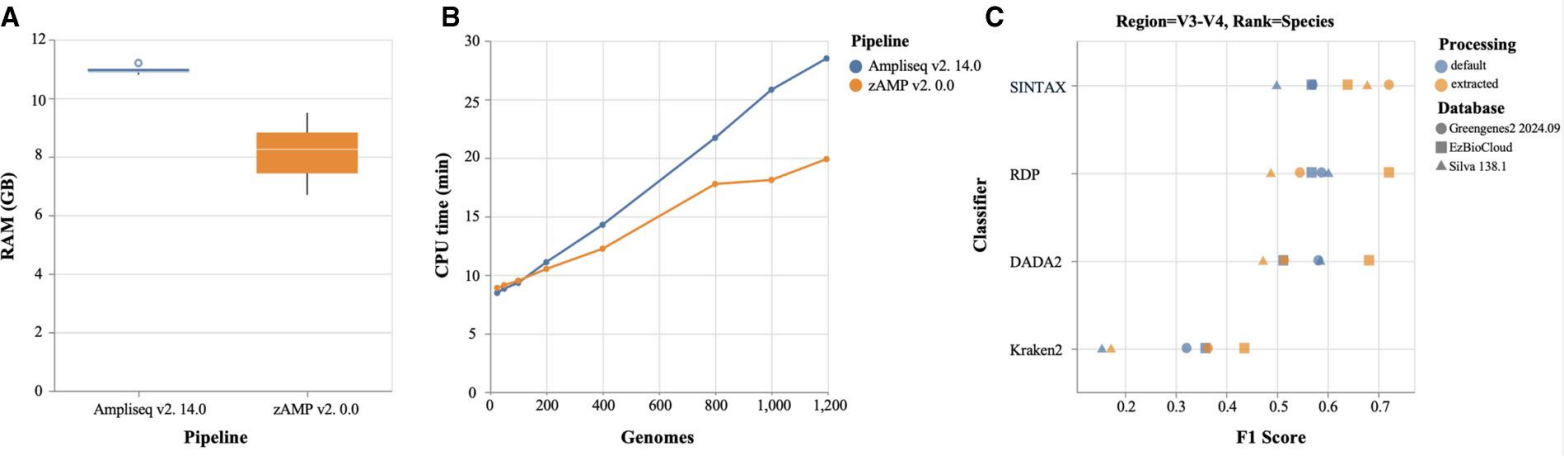
zAMP benchmarking results. (A) Physical memory usage in Gb between ampliseq and zAMP using the simulated subsets. (B) Comparison of process time (CPU time) in minutes and according to the number of genomes used in simulated samples analyzed by ampliseq and zAMP. (C) Comparison of Sintax, RDP, DADA2, and Kraken2 taxonomic assignment at species level, for the V3-V4 region colored according to the database type (default and extracted databases). Circles correspond to the Greengenes2 database, squares correspond to EzBioCloud, and triangles to the SILVA database.

In terms of computational efficiency, zAMP used less RAM (8 GB versus 10 GB) and ran faster compared to ampliseq (Fig. 2A and B). This gain likely reflects zAMP's per-sample denoising strategy versus ampliseq’s per-run approach. Regarding taxonomic assignment accuracy, both tools performed comparably with Greengenes2 database, yielding average F1-scores of 0.90 at genus level and 0.57 at species level across all subsets.

## 4 Secondary modules: database processing and *in silico* benchmarking

Popular 16S ribosomal RNA databases like Greengenes2 (McDonald *et al.* 2024) or SILVA (Quast *et al.* 2013) differ in taxonomy and contain inconsistently annotated taxa (Molano *et al.* 2024), such as species appearing in different genera (“convergent evolution”), duplicated names or empty ranks. To ensure compatibility with multiple taxonomic classifiers like RDP, zAMP detects “convergent evolution” and raises an error during database pre-processing (Fig. 1A). Duplicated names are removed and empty ranks are filled by propagating their nearest valid taxon (rank propagation), similar to RESCRIPt (Robeson *et al.* 2021). Currently, zAMP supports Greengenes2 (McDonald *et al.* 2024), SILVA (Quast *et al.* 2013), EzBioCloud (Yoon *et al.* 2017), UNITE (Abarenkov *et al.* 2024), and Eukaryome (Tedersoo *et al.* 2024) databases but accept any database in QIIME format.

As highlighted by QIIME 2’s feature-classifier plugin (Bokulich *et al.* 2018), training classifiers on primer-specific regions improves accuracy. Hence, zAMP can extract primers-specific regions from full length sequences with Cutadapt (Martin 2011). Extracted regions are then dereplicated with VSEARCH, and identical sequences are assigned a “merged taxonomy.” This is useful in a clinical setting where closely related bacteria causing similar clinical symptoms are commonly reported at the group level. For example, the *Staphylococcus aureus*, *schweitzeri* and *argenteus* species are all considered as *S. aureus* in clinical practice and cannot be distinguished using the widely used V3-V4 target sequence. Their taxonomy is merged into “*Staphylococcus aureus*/*schweitzeri/argenteus*.”

zAMP also incorporates an *in silico* module (Fig. 1C) to evaluate (i) whether selected PCR primers can amplify specific taxa of interest, (ii) the number of loci targeted by the primers on each genome, and (iii) taxonomic classification accuracy of the amplicons using different classifiers and databases. This *in silico* workflow downloads genomes from NCBI using assembly_finder (Chaabane *et al.* 2024), before simulating PCR amplicons with simulate-PCR (Gardner and Slezak 2014) or in-silico PCR (Ozer 2017). Each amplicon is then assigned a taxonomy using one or multiple classifiers (RDP, QIIME, DADA2, …) that is compared to the expected taxonomy. Hence, this workflow enables benchmarking the impact of primers, classifiers, and reference databases on taxonomic assignment across genomes in a reusable and flexible framework.

We assessed the performance of 16S rRNA primers targeting the V3–V4 subregion with three reference databases (SILVA 138.1, Greengenes2, and EzBiocloud) and four classification tools (RDP, DADA2, Sintax, and Kraken2) for taxonomic assignment accuracy (Fig. 2C). We also compared zAMP's processed database with default ones. For this, we created a mock community of 1196 pathogenic bacterial species using the *in silico* module. At the genus level, V3–V4 extracted and default databases performed similarly across classifiers, with a median F1 score of 0.85. Among the databases, Greengenes2 achieved the highest median score (F1 = 0.89), followed closely by SILVA (F1 = 0.86) and EzBiocloud (F1 = 0.83). Sintax was the top-performing classifier (F1 = 0.87), followed closely by RDP and DADA2 (both F1 = 0.86). At the species level, V3–V4 extracted databases improved average F1 scores only for some classifiers. Notably, Sintax performed best with the extracted Greengenes2 and SILVA 138.1 databases (F1 = 0.72 and F1 = 0.68, respectively), while RDP performed best with the extracted EzBioCloud database (F1 = 0.72). These results highlights the need to adapt database, classifier and primer choice to specific applications.

## 5 zAMPExplorer

Amplicon-based microbiota profiling produces complex datasets that require substantial bioinformatics and programming expertise. While zAMP automates upstream processing, interpreting community-level changes, visualizing results, and linking them to metadata remain a major barrier for researchers and clinicians. zAMPExplorer bridges this gap with a robust, user-friendly Shiny application that lets non-experts explore, analyze, and visualize microbiota data without coding. As a companion to zAMP, it accepts a phyloseq object as input and provides a comprehensive suite of downstream microbiota profiling tools. Leveraging multiple R packages, it automates key analyses and statistical evaluations, and provides intuitive, reproducible, and visually informative interpretation of microbial community data. zAMPExplorer supports the following range of downstream microbiota analysis:

- **Quality control and taxa overview**, including read-based rarefaction curves and Venn diagrams to assess sequencing depth and shared community members across groups.
- **Diversity and composition analysis** with alpha and beta diversity metrics, heatmaps, and ordination plots (e.g. PCoA, RDA) to investigate within- and between-sample variation. For example, clinicians can use diversity patterns to evaluate dysbiosis or recovery following treatment, while researchers can explore ecological gradients or host–microbiome associations.
- **Community typing** using Dirichlet Multinomial Modeling (DMM) to identify community structures (e.g. enterotypes) and link them to metadata.
- **Differential abundance testing** to identify taxa associated with environments, treatments or clinical factors.

**Figure 3.**
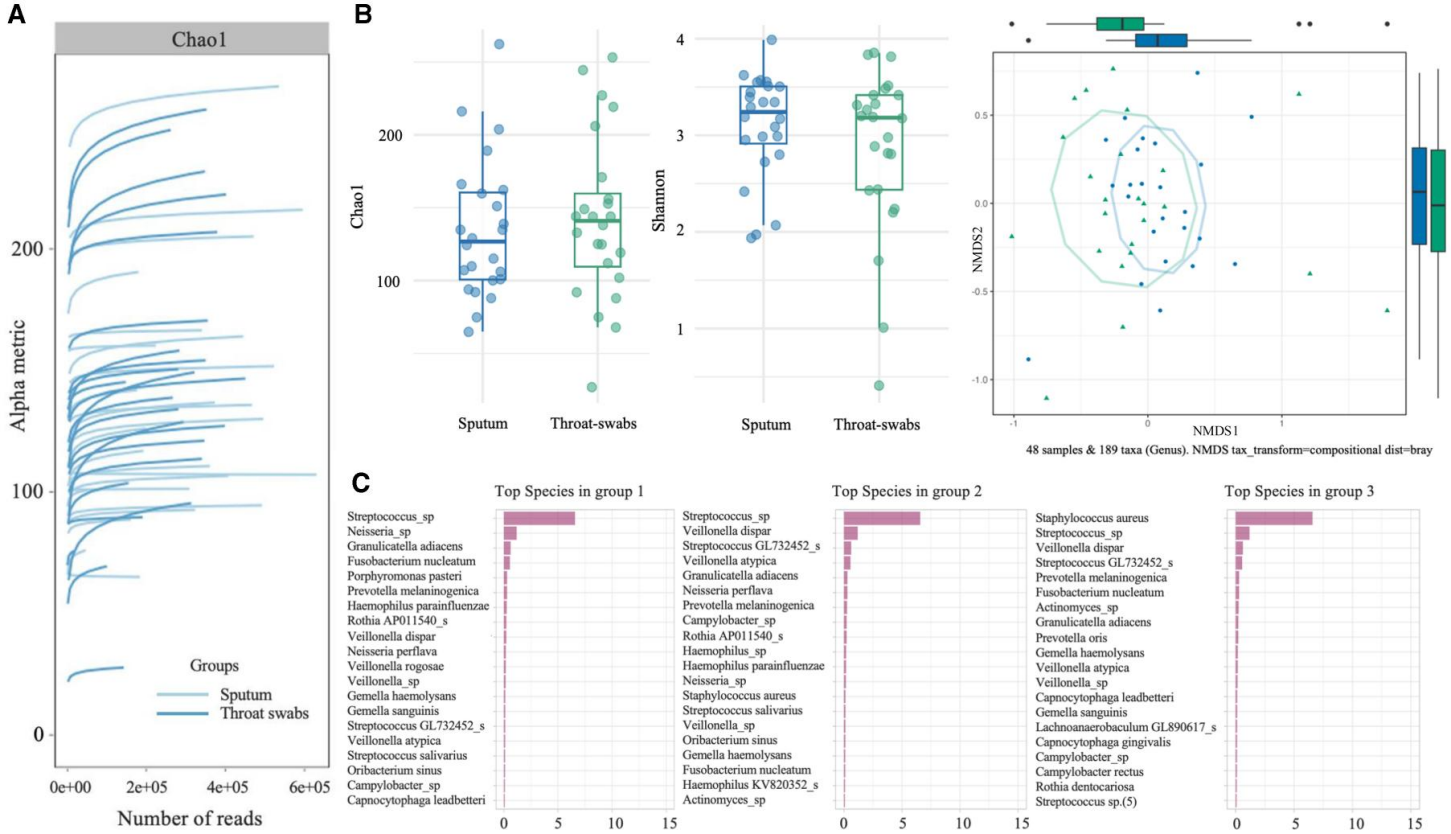
Use case of zAMPExplorer: microbiota profiling of paired sputum and throat-swab samples from cystic fibrosis pediatric patients. (A) Rarefaction curves display sequencing depth for each sample, with sputum and throat-swab groups, used to assess coverage adequacy for diversity analysis. (B) Alpha diversity is shown using boxplots for Chao1 (richness) and Shannon (evenness) indices by sample type. Beta diversity is visualized using NMDS ordination based on Bray–Curtis dissimilarity. Statistical overlays include PERMANOVA for group separation (*P*-value) and betadisper for dispersion estimation. (C) Barplots of top taxa per DMM-inferred community type. Each column displays the top 20 most abundant species within each of the three community types identified across all samples.

All modules produce reproducible, publication-ready outputs and foster exploratory analysis for hypothesis generation and interpretation of host–microbiota interactions. To showcase its utility, we applied zAMPExplorer to the MUCOVIB dataset (Nassirnia *et al.* 2025), comparing upper (throat-swab) and lower (sputum) airway microbiota in young children with cystic fibrosis (Fig. 3). Rarefaction curves confirmed comparable sequencing depth across samples, to capture microbiota diversity (Fig. 3A). Alpha and beta diversity assessments, evaluating within- and between-group variability, revealed comparable richness and evenness between sample types (Fig. 3B). NMDS ordination with PERMANOVA and dispersion testing showed a modest but significant compositional shift based on bacterial abundance (*P *= .001), despite considerable overlap. DMM modeling identified distinct community types driven by patient-specific factors rather than sample type (Fig. 3C). These results support the conclusion that throat swabs can capture relevant inter-individual microbial variation, reinforcing their value as a non-invasive alternative when sputum collection is not feasible. Designed for researchers and clinicians alike, zAMPExplorer combines core statistical methods with an interactive interface, enabling contextual interpretation of microbial community dynamics without prior bioinformatics expertise.

## 6 Conclusion

To streamline microbiome analysis, from raw data to visualization and statistical evaluation, we developed zAMP, an open-access pipeline for amplicon sequence data like 16S rRNA or ITS regions. Beyond standard processing, zAMP features a unique module for pre-processing user-defined databases, improving taxonomic assignment of closely related taxa, and an *in-silico* validation module to assess classification accuracy. To support downstream analyses, zAMPExplorer, an interactive RShiny application, enables quality control, diversity analysis, and differential abundance testing through intuitive data visualizations. Together, zAMP and zAMPExplorer form a comprehensive, reproducible, and user-friendly framework for microbiota profiling in both research and clinical contexts.

## Supplementary Material

vbaf255_Supplementary_Data

## Data Availability

The zAMP pipeline is available at https://github.com/metagenlab/zAMP. The zAMPExplorer Shiny application is available at https://github.com/metagenlab/zAMPExplorer. The Nextflow pipeline used to benchmark zAMP and other amplicon-based sequencing analysis pipelines is available at https://github.com/metagenlab/benchmark-zamp.
