# Supplementary material for: zAMP and zAMPExplorer: reproducible scalable amplicon-based metagenomics analysis and visualization
Source: Bioinform Adv. 2025 Nov 4;5(1):vbaf255. doi: 10.1093/bioadv/vbaf255 (PMC12603355; doi:10.1093/bioadv/vbaf255)
Supplement: vbaf255_Supplementary_Data [file vbaf255_supplementary_data.zip › Scherz_supplementary_data.docx]

**Supplementary data**

**zAMP and zAMPExplorer: Reproducible Scalable Amplicon-based Metagenomics Analysis and Visualization**

Valentin Scherz°, Sedreh Nassirnia°, Farid Chaabane°, Violeta Castelo-Szekely, Gilbert Greub, Trestan Pillonel, Claire Bertelli*

Institute of Microbiology, Lausanne University Hospital and University of Lausanne, Lausanne, Switzerland

° Equal contribution. * Corresponding author

***Supplementary Table 1. List of pathogens used for the benchmarking****. The list consists of bacterial species identified in the diagnostic setting at Lausanne University Hospital and indicates the genome accession used for the benchmarking of the different databases using the in-silico module.*

**Supplementary Table 2: Average precision, recall and F1-scores across genera and species for simulated reads analyzed by zAMP and ampliseq**

| **origin** | **rank** | **precision** | **recall** | **f1_score** |
| --- | --- | --- | --- | --- |
| nf-core/ampliseq | genus | 0.91 | 0.89 | 0.90 |
| nf-core/ampliseq | species | 0.60 | 0.54 | 0.57 |
| zAMP | genus | 0.91 | 0.89 | 0.90 |
| zAMP | species | 0.59 | 0.56 | 0.57 |
